# Orthorexia Nervosa: over concern or obsession about healthy food?

**DOI:** 10.1007/s40519-021-01110-x

**Published:** 2021-02-10

**Authors:** Caterina Novara, Susanna Pardini, Eleonora Maggio, Sofia Mattioli, Sara Piasentin

**Affiliations:** Department of General Psychology, University of Padova, Via Venezia 8, 35131 Padova, Italy

**Keywords:** Orthorexia Nervosa, Worry, Eating features, Obsessive–compulsive features, Dieting

## Abstract

**Purpose:**

Orthorexia Nervosa is characterized by specific behaviors frequently related to other psychopathological conditions, such as Obsessive–Compulsive Disorder (OCD) and Eating Disorders (EDs). Whereas ON can mainly be described as an excessive concern regarding healthy food, the study’s principal aim was to investigate if ON could be considered a condition related and differentiated from worry, other than OCD, EDs, perfectionism, anxiety, and depression.

**Method:**

To achieve these aims, 302 individuals from the general population were enrolled and were divided into two groups named “High EHQ” and “Low EHQ”, based on their Eating Habits Questionnaire’s score (EHQ-21).

**Results:**

Correlations of ON with EDs and non-adaptive perfectionism constructs emerged independently from Obsessive-Compulsive (OC) symptoms, and the same pattern was observed when comparing the High and the Low EHQ groups. The two groups also differ in the worry anxiety and depression constructs and are not affected by OC symptoms removal.

**Discussion:**

Our results confirm a relationship between ON with the typical ED, perfectionistic, anxious, and depressive symptomatology, mainly when the OC features are controlled; moreover, worry constructs could be considered characteristic of the ON phenomenology. This study does not entirely exclude the relationship with obsessive and compulsive characteristics, which could be associated with or serve as a mediator of the orthorexic behavior. Future research could explore the potential mediating or collateral role of OC symptoms.

**Level of evidence:**

Level III, evidence obtained from well-designed cohort or case–control analytic studies.

## Introduction

In 1997, Bratman coined the term Orthorexia Nervosa (ON), referring to a psychological condition characterized by excessive concern about eating healthy food with specific nutritional properties initially chosen to eat healthily, prevent and manage diseases including overweight. Healthy eating habits could also be like a pathological obsession. In this way, time spent to prepare and eat a meal becomes excessive [1–3] and could also relate to health problems (e.g., due to nutritional deficits) [2, 4, 5]. However, ON is still not classified as a psychopathological category by the Diagnostic and Statistical Manual of Mental Disorders-5 (DSM-5) [6]. The differences between a healthy eating style and ON are still unknown. The same goes for the differences between ON, Eating Disorders (EDs), and over concerns about healthy foods; therefore, research in this field is necessary.

To investigate ON and EDs relation and differences, several studies focused on specific demographic characteristics or nutritional choices, obtaining heterogeneous results about age [7–9], education level [10, 11], gender [7, 12, 13], and the Body Mass Index (BMI): most studies showed that BMI is unrelated to ON [8, 14, 15]. A relationship between high BMI and orthorexic tendencies has been highlighted [16, 17]. Otherwise, Arusoğlu et al. [18] found out that BMI could be an orthorexic tendency's predictor only in combination with EDs and Obsessive–Compulsive (OC) symptoms. Globally, discrepant results could be explained using different measuring instruments: most of these studies used the ORTO-15 [19] and its adapted versions. Indeed, these measures have psychometric limitations, such as the absence of a standardized sample and insufficient construct validity [20–22]. Besides, scholars also used different cut-offs to assess orthorexic symptomatology [20, 23]. ON's main features have not yet been identified due to conflicting results in the literature and ORTO-15 limitations. In the current study, the EHQ-21 [24] was used to assess ON as it exhibits good psychometric characteristics in different cultures [3, 23, 25].

## ON and Eating Disorders

ON shares some features with other recognized EDs, especially with Anorexia Nervosa (AN) and Bulimia Nervosa (BN) [11, 14, 26–32]. ON and AN/BN imply an over concern about food and lifestyle is modified by an excessive influence of diet's rigid adherence [2, 22, 31, 33]. Differently from ON, AN/BN are mainly focused on quantity rather than the quality of food, and body image disturbances and eating restrictions are driven by fears of gaining weight rather than healthy or ethical reasons [33]. In this regard, control could serve as a maintenance factor for strict eating habits. However, while individuals with AN/BN try to hide their behavior, individuals with ON show it off, exhibiting a superior attitude towards others [2], and may suffer from social isolation and somatic problems [2, 22, 33]. In a more recent study, Zickgraf et al. [31] confirmed ON's partial independence from EDs, emphasizing that the EHQ-21 “Problems” subscale can identify disordered eating habits different from not pathological healthy eating and from EDs.

## ON, worry and obsessive–compulsive symptoms

The literature highlighted relationships between OC symptoms and ON. The content of obsessions in ON usually concerns food and its calories, while rituals tend to be associated with research and preparation of those meals considered healthy in both patients with EDs [27] and the general population [15, 34]. Contaminations or harmful thoughts are the most frequent obsessions, whereas checking and washing rituals are the most frequent compulsions in ON [35, 36]. Moreover, ON features were only moderately associated with Obsessive–Compulsive Disorder (OCD) and EDs symptomatology in a sample of patients with Generalized Anxiety Disorder (GAD) and OCD; in this study, significant differences in ON severity between the two groups has not been shown [37]. Furthermore, in samples with EDs, the relationship between ED and OC symptomatology is confirmed [38, 39].

However, food obsessions prevalently are not ego-dystonic in individuals with ON [1, 33], and rituals could not intend to reduce anxiety symptoms related to thought, as it happens in OCD [40, 41]. Indeed, the repetitiveness and permanence of food-related thinking might suggest an excessive concern about the food healthiness and its preparation, rather than an obsession followed by a compulsion to neutralize it. Excessive attention or concern to prevent unhealthy food intake may lead to developing worries about a healthy, strict diet [37].

## ON, perfectionism, depression and anxiety features

Overlaps with the perfectionism dimensions were often found in populations with OC features or EDs [42]; they have also been linked to ON. A positive association between the maladaptive "Perfectionism" scale of the Eating Disorder Inventory-2 (EDI-2) [43] and greater ON tendencies has been shown [14]. Measured by the Multidimensional Perfectionism Scale (MPS) [44], a relation between perfectionism and orthorexic tendencies in a sample of university students emerged [3, 36]; moreover, a relationship between the "Concern Over Mistakes" MPS subscale and ON tendencies has been highlighted [45]. Symptoms of depression and anxiety are often found positively associated with ON characteristics [23, 46], as well as with OCD and ED symptoms [47, 48], although in the literature related to ON, opposite results came to light [50].

Scientific literature put in evidence a heterogeneous pattern of features implied in the ON conceptualization with an overlap between EDs (especially, AN and BN) and strong relationships with OC [32, 49, 50] and perfectionism features [3, 14, 32, 36, 45]. To date, while relations between these characteristics are quite evident, associations with worry and independence from obsessions and compulsions have been less investigated.

Based on our knowledge, this is the first study investigating the relationship between ON and worry and its independence from OC symptomatology. Indeed, the study's principal aim was to examine if ON could be considered a disorder independent from OCD and related to worry's content and cognitive processes, controlling the Obsessive-Compulsive Inventory-R effect. Moreover, other aims were to compare ED, perfectionistic anxiety, and depression features considering two groups of individuals with High and Low EHQ scores, respectively. It is expected that removing OC features, relationships between ON and ED, perfectionism, anxiety, and depression characteristics would become more evident.

## Methods

### Participants

The study group consisted of 302 students recruited from the university student population in Northern Italy, using snowball sampling. The first small group of students provided the contacts of other university students who were subsequently contacted for participation in the research. Initially, data were collected from 310 participants, but eight individuals were excluded from the analysis because of exclusion criteria (presence of mental or neurological disease) identified using a demographic schedule.

The final sample was divided into two groups named, respectively, “High EHQ” (*n* = 43) (it included those who scored higher than 50 on the Eating Habits Questionnaire; corresponding to the 90th percentile based on the total sample scores) and “Low EHQ” (*n* = 259) (EHQ total raw score < 50). 48.8% of the first group were female (*n* = 21), with a mean age of 20.60 years (SD = 2.44; ranging from 18 to 31 years) and a mean of 14.81 (1.23) years of education. This group had a BMI mean of 21.44 (SD = 2.02). As far as the “Low EHQ” group is concerned, 58.5% were female, with a mean age of 20.83 (SD = 3.33; ranging from 18 to 49 years), a mean of 14.90 (SD = 1.58) years of education, and a BMI mean of 21.30 (SD = 2.56). Considering the demographic variables age, BMI, and years of education, no statistically significant differences between the two groups were found (Fisher *F* ranging from 0.17 to 0.12; n.s.), and the same occurred considering the variable gender ($\chi^{2}$ = 1.39; n.s.).

Individuals participated voluntarily and gave their written consent before taking part in the study. Participants were fully informed about the research; moreover, the anonymity and the confidentiality of the collected data were guaranteed.

They responded to a series of paper-and-pencil self-report questionnaires in a single session that lasted approximately 50 min. All measures were administered in a counterbalanced order to avoid any order effects.

This study was conducted according to the Declaration of Helsinki and was approved by the Department of General Psychology’ Ethical Committee, University of Padova.

## Measures

Demographic schedule: participants were asked to complete a demographic schedule to collect data about gender, age, education levels, and BMI. Any present mental disorder or neurological disease was investigated as exclusion criteria.

The Eating Habits Questionnaire (EHQ-21) [24]; the Italian version by [23], is a 21-item self-report questionnaire used to assess Orthorexia Nervosa’s features on a four-point Likert scale (“False, Not at All True”, “Slightly True”, “Mainly”). The items are grouped into three subscales named “Knowledge” (it refers to knowledge about healthy eating), “Feelings” (it concerns feelings, emotions, and sensations related to following a healthy diet), and “Problems”. The internal consistency (0.82 < Cronbach’s *α* < 0.90) and test–retest reliability (0.72 < *r* < 0.81) are good.

Psychometric properties of the EHQ-21 within the Italian population were confirmed by an exploratory and confirmatory factorial analysis that put in evidence the original EHQ structure. Moreover, a good internal consistency and a one-month test–retest reliability were highlighted (*r* ranging from 0.50 to 0.75; 0.001 < *p* < 0.01) [23].

The current study showed good internal consistency (Total score: Cronbach’s *α* = 0.89; “Knowledge”: Cronbach’s *α* = 0.79; “Problems”: Cronbach’s *α* = 0.86), except for the “Feelings” subscale, where Cronbach’s *α* is acceptable (Cronbach’s *α* = 0.68).

The Eating Disorder Inventory-3 (EDI-3) [51]; the Italian version by [52], is a 91-item self-report questionnaire used to assess both symptoms and psychological-related eating disorders’ features. Items are scored on a six-point Likert scale (“Always”, “Usually”, “Often”, “Sometimes”, “Rarely”, and “Never”). Specifically, the questionnaire is composed of twelve subscales: three measuring eating disorder symptoms (“Drive for Thinness”, “Bulimia”, and “Body Dissatisfaction”), and nine investigating different general psychological features considered as some of the risk factors for the development of an EDs (“Low Self-Esteem”, “Personal Alienation”, “Interpersonal Insecurity”, “Interpersonal Alienation”, “Interoceptive Deficits”, “Emotional Dysregulation”, “Perfectionism”, “Asceticism”, and “Maturity Fears”). The EDI-3 also yields the “Risk for Eating Disorders” (EDI-RISK), a score obtained considering the “Drive for Thinness”, “Bulimia”, and “Body Dissatisfaction” subscales. Its internal consistency (0.90 < Cronbach’s *α* < 0.97) and test–retest reliability (*r* = 0.98) were excellent in the validation sample represented by EDs patients. Moreover, the Italian version displayed a good internal consistency (0.72 < Cronbach’s *α* < 0.94).

For the current study, Cronbach’s *α* displayed a good internal consistency. The “Asceticism” subscale was considered acceptable (Cronbach’s *α* = 0.60) [53] after “item 88” had been excluded.

The Obsessive Compulsive Inventory-Revised (OCI-R) [54]; the Italian version by [55], is an 18-item self-report questionnaire used to assess OCD symptoms on a five-point Likert scale. The questionnaire is composed of six subscales: “Washing”, “Ordering”, “Hoarding”, “Mental Neutralizing”, “Obsessing”, and “Checking”. Regarding the Italian version, the confirmatory factor analysis proved the original six-factors structure and a good test–retest reliability (0.76 < *r* < 0.99).

The current study highlighted good internal consistency (Table 1).

**Table 1:** Correlations and partial correlations between the Eating Habits Questionnaire and the demographic features and questionnaires

	EHQ-Total	EHQ-Total W/O OCI-R Total	EHQ-P	EHQ-P. W/O OCI-R Total	EHQ-K	EHQ-K. W/O OCI-R Total	EHQ-F	EHQ-F W/O OCI-R Total	Cronbach’s Alpha
Age	− 0.02 ns		− 0.04 ns		− 0.03 ns		0.04 Ns		
BMI	0.06 ns		0.08 ns		0.01 ns		0.04 ns		
EDI-3									.95
Drive for Thinness	.35 < 0.001	.30 < 0.001	.36 < 0.001	.29 < 0.001	.16 < 0.01	.14 < 0.05	.31 < 0.001	.30 < 0.001	.82
Bulimia	.22 < 0.001	.20 < 0.01	.25 < 0.001	.22 < 0.001	.06 ns	.07 ns	.18 < 0.01	.19 < 0.01	.82
Body Dissatisfaction	.11 ns	.04 ns	.10 ns	.02 ns	.00 ns	− .04 ns	.18 < 0.01	.15 < 0.05	.86
EDI-Risk	.26 < 0.001	.19 < 0.01	0.26 < 0.001	.18 < 0.01	0.08 ns	.05 ns	0.28 < 0.001	.24 < 0.001	
Low Self-Esteem	.00 ns	− .04 ns	.09 ns	.04 ns	− .16 < 0.01	− .17 < 0.01	.01 ns	.00 ns	.84
Personal Alienation	.10 ns	.02 ns	.21 < 0.001	.16 < 0.05	− .11 < 0.05	− .12 ns	.07 ns	− .02 ns	.72
Interpersonal Insecurity	.09 ns	.02 ns	.19 < 0.01	.12 ns	− .06 ns	− .11 ns	.03 ns	− .02 ns	.80
Interpersonal Alienation	.14 < 0.05	.05 ns	.23 < 0.001	.14 < 0.05	− .01 ns	− .08 ns	.04 ns	− .01 ns	.73
Interoceptive Deficits	.24 < 0.001	.20 < 0.01	.31 < 0.001	.23 < 0.001	.04 ns	.04 ns	.20 < 0.01	.20 < 0.001	.82
Emotional Dysregulation	.16 < 0.01	.10 ns	.22 < 0.001	.15 < 0.05	.00 ns	− .02 ns	.11 ns	.08 ns	.77
Perfectionism	.25 < 0.001	.18 < 0.01	.21 < 0.001	.10 ns	.21 < 0.001	.19 < 0.01	.19 < 0.01	.17 < 0.01	.71
Asceticism	.36 < 0.001	.32 < 0.001	.38 < 0.001	.30 < 0.001	.19 < 0.01	.19 < 0.01	.27 < 0.001	.28 < 0.001	.60
Maturity Fears	.07 ns	.06 ns	.16 < 0.01	.13 < 0.05	− .09 ns	− .07 ns	.04 ns	.04 ns	.77
OCI-R									
OCI-R Total	.31 < 0.001		.36 < 0.001		.12 < 0.05		.21 < 0.001		.91
OCI-R Washing	.32 < 0.001		.35 < 0.001		.17 < 0.01		.22 < 0.001		.76
OCI-R Checking	.27 < 0.001		.32 < 0.001		.12 < 0.05		.17 < 0.01		.71
OCI-R Ordering	.24 < 0.001		.27 < 0.001		.12 < 0.05		.17 < 0.01		.84
OCI-R Obsessing	.25 < 0.001		.27 < 0.001		.11 ns		.20 < 0.01		.80
OCI-R Hoarding	.18 < 0.01		.24 < 0.001		.03 ns		.10 ns		.73
OCI-R Mental Neutralizing	.13 < 0.05		.20 < 0.01		− .02 ns		.08 ns		.76
WDQ									
WDQ Total	.20 < 0.001	.12 ns	.24 < 0.001	.13 < 0.05	− .02 ns	− .06 ns	.26 < 0.001	.22 < 0.001	.94
WDQ Relationship	.09 ns	.04 ns	.14 < 0.05	.06 ns	− .07 ns	− .09 ns	.15 < 0.01	.13 < 0.05	.82
WDQ Lack of Confidence	.15 < 0.05	.08 ns	.17 < 0.01	.08 ns	− .03 ns	− .06 ns	.21 < 0.001	.18 < 0.01	.86
WDQ Aimless Future	.16 < 0.01	.08 ns	.19 < 0.01	.08 ns	− .02 ns	− .06 ns	.21 < 0.001	.18 < 0.01	.77
WDQ Work Incompetence	.26 < 0.001	.17 < 0.01	.29 < 0.001	.18 < 0.01	.05 ns	.02 ns	.26 < 0.001	.23 < 0.001	.75
WDQ Financial	.21 < 0.001	.11 ns	.23 < 0.001	.12 < 0.05	.02 ns	− .04 ns	.25 < 0.001	.19 < 0.01	.69
PSWQ	.15 < 0.01	.08 ns	.15 < 0.01	.06 ns	.00 ns	− .03 ns	.22 < .0.001	.20 < 0.01	.92
MPS									
MPS Total	.37 < 0.001	.28 < 0.001	.30 < 0.001	.18 < 0.01	.28 < 0.001	.26 < 0.001	.33 < 0.001	.28 < 0.001	.93
MPS Personal Standard	.34 < 0.001	.31 < 0.001	.23 < 0.001	.18 < 0.01	.35 < 0.001	.34 < 0.001	.30 < 0.001	.27 < 0.001	.83
MPS Concern over Mistakes	.28 < 0.001	.20 < 0.001	.25 < 0.001	.12 < 0.05	.18 < 0.01	.17 < 0.01	.24 < 0.001	.22 < 0.001	.90
MPS Doubting of Action	.32 < 0.001	.27 < 0.001	.31 < 0.001	.23 < 0.001	.16 < 0.01	.15 < 0.05	.31 < 0.001	.29 < 0.001	.77
MPS Parental Expectations	.23 < 0.001	.16 < 0.05	.21 < 0.001	.09 ns	.14 < 0.05	.14 < 0.05	.22 < 0.001	.20 < 0.01	.88
MPS Parental Criticism	.21 < 0.001	.12 ns	.24 < 0.001	.13 < 0.05	.08 ns	.05 ns	.16 < 0.01	.10 ns	.74
MPS Organization	.13 < 0.05	.07 ns	.07 ns	.01 ns	.18 < 0.05	.15 < 0.05	.09 ns	.05 ns	.89
BAI	.13 < 0.05	.04 ns	.14 < 0.05	.01 ns	.00 ns	− .04 ns	.18 < 0.05	.15 < 0.05	.90
BDI-II	.10 ns	.01 ns	.16 < 0.01	.04 ns	− .04 ns	− .06 ns	.07 ns	.02 ns	.90

*EHQ-2*1 Eating Habits Questionnaire; *EHQ-P* Eating Habits Questionnaire-Problems; *EHQ-K* Eating Habits Questionnaire-Knowledge; *EHQ-F* Eating Habits Questionnaire-Feelings; *EDI-3* Eating Disorder Inventory-3; *EDI-Risk* Risk for Eating Disorder considering the “Drive for Thinness”, “Bulimia” and “Body Dissatisfaction” subscales; *OCI-R* Obsessive Compulsive Inventory-*Revised;* *WDQ* Worry Domains Questionnaire; *PSWQ* Penn State Worry Questionnaire; *MPS* Multidimensional Perfectionism Scale; *BAI* Beck Anxiety Inventory; *BDI-II* Beck Depression Inventory-II; *W/O* Controlling for the OCI-R effect

The Beck Anxiety Inventory (BAI) [56]; the Italian version by [57], is a 21 item self-report questionnaire used to assess the severity of anxiety symptoms, with a four-point Likert scale. Both the original and the Italian versions showed good internal consistency (Cronbach’s *α* = 0.92 and 0.89, respectively) and adequate test–retest reliability. The current study showed good internal consistency (Table 1).

The Beck Depression Inventory-Second Edition (BDI-II) [58]; the Italian version by [59], is a 21-item self-report questionnaire used to assess the severity of depression symptoms, and it is rated on a four-point Likert scale. The original and the Italian versions showed an excellent internal consistency (Cronbach’s *α* = 0.93 and 0.87, respectively) and test–retest reliability (*r* = 0.93 and 0.76, respectively). In the current study, the questionnaire proved to have good internal consistency (Table 1).

The Penn State Worry Questionnaire (PSWQ) [60]; the Italian version by [61], is a 16-item self-report questionnaire based on a five-point Likert scale that assesses the dimensions of traits such as generality, excessiveness, and uncontrollability of pathological worry. Its internal consistency was good (original version: Cronbach's *α*= 0.93; Italian version *α* = 0.85). The current study highlighted excellent internal consistency (Table 1).

The Worry Domains Questionnaire (WDQ) [62, 63]; the Italian version by [61], is a 25-item self-report questionnaire used to assess the specific content of worry. It is divided into five subscales: “Relationship”, “Lack of Confidence”, “Aimless Future”, “Work”, and “Financial”. Its internal consistency was excellent (original version Cronbach’s *α* = 0.92 and Italian version 0.65 < Cronbach’s *α* < 0.90).

The current study showed good internal consistency (Table 1).

The Multidimensional Perfectionism Scale (MPS) [44, 64]; the Italian version by [65], is a 35-item self-report questionnaire rated on a five-point Likert scale. It is administered to assess different traits of perfectionism that are operationalized in six subscales: “Concern over Mistakes”, “Personal Standards”, “Parental Expectations”, “Parental Criticism”, “Doubting of Actions”, and “Organization”. The original and the Italian internal consistency are good (0.76 < Cronbach’s *α* < 0.93). In the current study, all subscales proved to have good internal consistency (Table 1).

### Data analysis

All analyses were carried out using the SPSS Statistics-Version 22. First of all, Cronbach’s alpha was investigated for all the administered self-report questionnaires.

Correlation and partial correlation analysis were used to investigate the relationship between the EHQ and other questionnaires. Multivariate ANOVA, and ANCOVA, and a Chi-squared index were used to explore the differences between groups.

## Results

### Correlations with age and BMI

No correlations were found between the EHQ total/subscales’ scores and, respectively, age and BMI.

### Correlations with ED characteristics

Positive correlations between the EHQ total score and the EDI-3 subscales (“Drive for Thinness”, “Bulimia”, “EDI-RISK”, “Interoceptive Deficits”, “Perfectionism”, and “Asceticism” subscales) were highlighted (0.22 < *r* < 0.36; *p* < 0.001). The same pattern of correlations was found for the EHQ “Problems” subscale and the EDI-3 subscales (0.25 < *r* < 0.38; *p* < 0.001) and less strongly with “Maturity Fears” (*r* = 0.16; *p* < 0.01), “Interpersonal Insecurity” (*r* = 0.19; *p* < 0.01), “Emotional Dysregulation” (*r* = 0.22; *p* < 0.001), “Interpersonal Alienation” (*r* = 0.23; *p* < 0.001), and “Personal Alienation” (*r* = 0.21; *p* < 0.001). The EHQ “Knowledge” subscale is positively correlated only with the EDI-3 “Perfectionism”, “Asceticism”, and “Drive for Thinness” subscales (0.16 < *r* < 0.21; *p* < 0.01) while it is negatively correlated with the “Low Self-Esteem” subscale (*r* = − 0.16; *p* < 0.01). Finally, positive correlations between the EHQ “Feelings” subscale and the EDI-3 “Drive for Thinness”, “Bulimia”, “Body Dissatisfaction”, “EDI-RISK”, “Interoceptive Deficits”, “Perfectionism”, “Asceticism” subscales were found (0.18 < *r* < 0.31; *p* < 0.01). All these relations remained significant if the OCI-R effect was controlled except for the “Interpersonal Insecurity” and “Perfectionism” subscales and the EHQ “Problems” subscale.

### Correlations with OC symptoms

Correlations were also put in evidence between the EHQ total and the OCI-R Total and subscales (0.13 < *r* < 0.31; *p* < 0.05). The EHQ “Problems” subscale shown positive relations with all the OCI-R subscales and total score (0.20 < *r* < 0.36; *p* < 0.01), the EHQ “Knowledge” with the OCI-R “Total/Washing/Checking/Ordering” (0.12 < *r* < 0.17; *p* < 0.05) and the EHQ “Feelings” subscale with the OCI-R “Total/Washing/Checking/Ordering/Obsessing” subscales (0.17 < *r* < 0.22; *p* < 0.01).

### Correlations with worry

Positive relations between the EHQ total score and EHQ “Problems” subscale with the WDQ Total and subscales are highlighted (0.14 < *r* < 0.29; *p* < 0.05) (0.15 < *r* < 0.26; *p* < 0.05) except for the “Relationship” subscale. The same pattern of correlations regards the EHQ “Problems” subscale (0.14 < *r* < 0.29; *p* < 0.05), and the majority of the subscales related are affected by the OCI-R elimination. The EHQ “Feelings” subscale correlates with the total and subscales of the WDQ (0.15 < *r* < 0.26; *p* < 0.01), and none of these were affected by the OCI-R removal, while the EHQ “Knowledge” subscale did not correlate with any worry constructs.

The PSWQ has a weak correlation with the EHQ total and “Problems” subscale (*r* = 0.15 for both *p* < 0.01) and is affected by the OCI-R removal, but the EHQ “Feelings” has a stronger relationship with worry’s subscale (*r* = 0.22; *p* < 0.001) that remains after removal. No relation emerged with the EHQ “Knowledge”.

### Correlations with perfectionism

The EHQ Total is positively related with the MPS Total, “Personal Standard”, “Concern over Mistakes”, “Doubting of Action”, and “Parental Expectations” subscales (0.23 < *r* < 0.37; *p* < 0.001) and remains still significant after removing the effect of the OCI-R. Other subscales have weaker correlations and are much more affected by the OCI-R removal.

### Correlations with anxiety and depression

Weak or not significant correlations emerged with the BAI and the BDI-II except for the EHQ “Feelings” subscale and the BAI (*r* = 0.18; *p* < 0.05) that remain significant after the OCI-R removal.

### Differences between the “High-EHQ” and the “Low-EHQ” groups

Differences in psychological constructs between the two EHQ groups have been highlighted. Specifically, the “High EHQ” individuals obtained, on average, greater scores compared to the other group considering the EDI-3 and its subscales (“Risk”, “Drive for Thinness”, “Bulimia”, “Personal Alienation”, “Interpersonal Insecurity”, “Interpersonal Alienation”, “Interoceptive Deficits”, “Emotional Dysregulation”, “Asceticism”; 5.45 < *F* < 28.03; *p* < 0.05). These results remained still significant after controlling for the OCI-R effect (10.95 < *F* < 23.58; *p* < 0.00). The scales of “Body Dissatisfaction”, “Low Self-Esteem”, “Perfectionism” and “Maturity Fears” became significant only after removing OCI-R (5.40 < *F* < 13.27; *p* < 0.01).

The “High EHQ” group differs from the “Low EHQ” one in the OCI-R total and its subscales except for the “Checking” and the “Mental Neutralizing” scales (4.59 < *F* < 7.70; *p* < 0.03). Groups differ in the content of worry measured through the WDQ (5.77 < *F* < 9.89; *p* < 0.02; 24.46 < *F* < 36.12; *p* < 0.00) with the “Relationship” and “Lack of Confidence” subscales, if the OCI-R total is in covariate (17.07 < *F* < 27.24; *p* < 0.00), and in the process of worry measured through the PSWQ that become significant after the removal of the OCI-R (*F* = 17.59; *p* < 0.00).

Groups differ in the MPS total and its subscales “Personal Standard”, “Concern over Mistakes”, and “Doubting of Action” subscales (5.05 < *F* < 14.69; *p* < 0.03; controlling for the OCI-R: 7.52 < *F* < 30.82; *p* < 0.00); differences regarding “Parental Expectation” and “Parental Criticism” emerged only after controlling for the OCI-R (10.42 < *F* < 13.79; *p* < 0.00). Finally, as for the “Organization” subscale, no difference between the groups was found.

The BAI and the BDI-II were found to be different between the two groups only after removing the OCI-R (19.78 < *F* < 22.25; *p* < 0.00) (Table 2).

**Table 2:** Comparisons between the High and the Low EHQ groups

	High EHQ *N* = 37	Low EHQ *N* = 225	*F*(_d.f.)_/OCI-R TOTAL in covariate(_d.f.)_	*p* value OCI-R TOTAL in covariate	Partial Eta squared/OCI-R TOTAL in covariate
EDI Risk Mean (SD)	27.73 (20.43)	20.44 (17.11)	5.45_(1)_/11.11_(2)_	0.02/0.001	0.021/0.08
Drive for Thinness Mean (SD)	9.14 (7.33)	5.07 (6.01)	13.66_(1)_/15.10_(2)_	0.001/0.001	0.05/0.10
Bulimia Mean (SD)	6.95 (6.33)	4.04 (4.73)	10.86_(1)_/10.95_(2)_	0.001/0.001	0.04/0.08
Body Dissatisfaction Mean (SD)	11.65 (9.57)	11.34 (9.09)	0.04_(1)_/5.40_(2)_	ns/0.01	−/0.04
Low Self-Esteem Mean (SD)	5.27 (4.62)	4.90 (4.79)	0.19_(1)_/6.90_(2)_	ns/0.001	−/0.05
Personal Alienation Mean (SD)	7.08 (4.61)	5.06 (4.28)	6.95_(1)_/17.10_(2)_	0.01/0.001	0.026/0.12
Interpersonal Insecurity Mean (SD)	10.70 (5.86)	7.44 (5.64)	10.51_(1)_/12.65_(2)_	0.001/0.001	0.039/0.09
Interpersonal Alienation Mean (SD)	9.05 (4.99)	6.35 (4.52)	11.06_(1)_/16.75_(2)_	0.001/0.001	0.04/0.12
Interoceptive Deficits Mean (SD)	9.62 (6.86)	5.97 (5.71)	12.27_(1)_/23.58_(2)_	0.001/0.001	0.045/0.15
Emotional Dysregulation Mean (SD)	6.73 (5.36)	4.15 (4.73)	9.99_(1)_/14.31_(2)_	0.002/0.001	0.037/0.10
Perfectionism Mean (SD)	7.86 (3.79)	6.51 (4.45)	3.05_(1)_/13.27_(2)_	ns/0.001	−/0.09
Asceticism Mean (SD)	7.95 (4.48)	4.45 (3.58)	28.03_(1)_/21.46_(2)_	0.001/0.001	0.10/0.14
Maturity Fears Mean (SD)	10.68 (6.21)	8.98 (5.82)	2.65_(1)_/7.45_(2)_	ns/0.001	−/0.05
OCI Total Mean (SD)	17.30 (13.93)	11.63 (11.07)	7.70_(1)_	0.006	0.029
OCI Washing Mean (SD)	2.95 (3.33)	1.52 (2.14)	11.78_(1)_	0.001	0.043
OCI Checking Mean (SD)	2.59 (2.70)	1.81 (2.28)	3.53_(1)_	ns	0.013
OCI Ordering Mean (SD)	3.59 (3.47)	2.39 (2.79)	5.50_(1)_	0.02	0.021
OCI Obsessing Mean (SD)	3.68 (3.28)	2.57 (2.85)	4.59_(1)_	0.03	0.017
OCI Hoarding Mean (SD)	3.35 (3.15)	2.28 (2.53)	5.27_(1)_	0.023	0.02
OCI Mental Neutralizing Mean (SD)	1.14 (1.65)	1.05 (1.97)	0.06_(1)_	ns	0.000
WDQ Total Mean (SD)	42.51 (19.01)	34.05 (17.87)	7.00_(1)_/36.12_(2)_	0.01/0.001	0.03/0.22
WDQ Relationship MEAN (SD)	7.54 (4.17)	6.62 (4.72)	1.24_(1)_/17.08_(2)_	ns/0.001	0.005/0.12
WDQ Lack of Confidence Mean (SD)	8.81 (4.77)	7.27 (4.62)	3.52_(1)_/27.24_(2)_	ns/0.001	0.01/0.17
WDQ Aimless Future Mean (SD)	9.08 (4.59)	7.28 (3.72)	5.77_(1)_/26.71_(2)_	0.02/0.001	0.02/0.17
WDQ Work Incompetence Mean (SD)	9.35 (4.59)	7.20 (3.72)	9.89_(1)_/24.46_(2)_	0.002/0.001	0.04/0.16
WDQ Financial Mean (SD)	7.73 (4.75)	5.67 (4.04)	7.82_(1)_/25.87_(2)_	0.01/0.001	0.03/0.17
PSWQ Mean (SD)	47.86 (13.08)	44.48 (12.04)	2.45_(1)_/17.59_(2)_	ns/0.001	−/0.12
MPS Total Mean (SD)	103.46 (17.99)	93.42 (20.57)	7.83_(1)_/23.04_(2)_	0.006/0.001	0.03/0.15
MPS Personal Standard Mean (SD)	24.62 (5.44)	21.49 (5.59)	10.04_(1)_/7.52_(2)_	0.002/0.001	0.04/0.06
MPS Concern over Mistakes Mean (SD)	25.51 (7.51)	22.58 (7.33)	5.05_(1)_/19.88_(2)_	0.03/0.001	0.02/0.13
MPS Doubting of Action Mean (SD)	12.49 (3.31)	10.24 (3.31)	14.69_(1)_/30.82_(2)_	0.001/0.001	0.05/0.19
MPS Parental Expectations Mean (SD)	10.92 (3.94)	10.40 (4.64)	0.41_(1)_/10.42_(2)_	ns/0.001	−/0.07
MPS Parental Criticism Mean (SD)	8.30 (2.92)	7.76 (3.17)	0.93_(1)_/13.79_(2)_	ns/0.001	−/0.10
MPS Organization Mean (SD)	21.62 (4.44)	20.95 (5.20)	0.55_(1)_/1.29_(2)_	ns/ns	−/−
BAI Mean (SD)	12.62 (10.26)	11.12 (9.00)	0.86_(1)_/22.25_(2)_	ns/0.001	−/0.15
BDI-II Mean (SD)	9.73 (7.21)	8.04 (8.06)	1.44_(1)_/19.78_(2)_	ns/0.001	−/0.13

*EHQ-21* Eating Habits Questionnaire; *EHQ-P* Eating Habits Questionnaire-Problems; *EHQ-K* Eating Habits Questionnaire-Knowledge; *EHQ-F* Eating Habits Questionnaire-Feelings; *EDI-3* Eating Disorder Inventory-3; *EDI-Risk* Risk for Eating Disorder considering the “Drive for Thinness”, “Bulimia” and “Body Dissatisfaction” subscales; *OCI-R* Obsessive Compulsive Inventory-*Revised;* *WDQ* Worry Domains Questionnaire; *PSWQ* Penn State Worry Questionnaire; *MPS* Multidimensional Perfectionism Scale; *BAI* Beck Anxiety Inventory; *BDI-II* Beck Depression Inventory-II; *d.f.* degrees of freedom; *SD* Standard Deviation

## Discussion

Overall, the present study aimed to investigate the relationship between the core features of Orthorexia Nervosa and other psychological constructs. Specifically, one of the aims of this study was to investigate ON’s relations with both content and cognitive processes of worry controlling for the OCI-R effects to understand if ON could be considered independent from OC symptomatology. Moreover, other aims were to compare OC, ED, perfectionistic, anxiety, and depression features considering two groups of individuals with High and Low EHQ scores, respectively.

Our results confirm a relationship between ON with ED symptomatology [11, 14, 26–32]. Particularly considering the High-EHQ group when the OC features are controlled. The EHQ subscales are related to the risk of having an Eating Disorder. Specifically, with the constructs of “Drive for Thinness” and “Bulimia”, “Interoceptive Deficits”, “Perfectionism”, and “Asceticism”, highlighting and confirming the nuclear factors which combined ON and ED [11, 14, 26–32]. Specifically, the EHQ “Problems” subscale is also related to the “Personal Alienation”, “Interpersonal Alienation”, “Emotional Dysregulation”, and “Maturity Fears” scales confirming the relationship with EDs’ dysfunctional aspects. It is interesting to note that relations with ED features, despite a reduction, remain when the OCI-R total score is controlled, underlining ON’s independence from OCD characteristics. Differences between the High and Low EHQ features showed the same trend: all the subscales related to eating disorder symptomatology result to differentiate.

Moreover, some subscales (“Body Dissatisfaction”, “Low Self-Esteem”, “Maturity Fears”, and “Perfectionism”) differentiated the groups but only if the OCI-R is covariate, showing that OC features could be considered a confounding factor in the relation between ON and EDs.

Weak relations between ON and worry's contents (assessed by the WDQ) and worry processes (assessed by the PSWQ) have been highlighted and, in general, are affected by the OCI-R removal. Instead, comparisons between the High and Low EHQ groups show significant differences for all the WDQ subscales and the PSWQ that become greater if the OCI-R effect is controlled. Moreover, an increase in eta squared has emerged, highlighting that worry’s contents and processes could be considered characteristic of the ON phenomenology. Globally, it is important to highlight how the EHQ “Feelings” subscale is the only one not to be affected by the OCI-R effect in all the correlations, both considering the WDQ and the PSWQ that highlight how ON’s emotions are connected with contents and processes of worry rather than OC symptomatology.

As in other studies, obsessive symptoms are positively related both with the EHQ “Total” and “Problems” subscale, showing a partial overlap between constructs (particularly for the symptomatology’s severity) [33]. Instead, the “Knowledge” and “Feelings” EHQ subscales are less strongly related, and only with the OCI-R “Washing”, “Checking” and “Ordering” subscales that are those that are usually found in samples of individuals with EDs [39, 66]. Finally, only the EHQ “Feelings” subscale is related to the OCI-R “Obsessing” scale highlighting how ON emotions are connected to repetitive thought processes. Moreover, the High and Low EHQ groups comparison has been put in evidence significant differences considering the OCI-R total and subscales, except for the “Mental Neutralizing” scale.

This mixed pattern could be explained, considering that ON and EDs are not different in the type of process involved as they are in the content. Indeed, ON is similar to EDs because both focus on food and ego-syntonicity, but they might have different aims concerning healthy food and weight control [22]. ON processes could be similar to the worry processes, but they may be different from OCD functioning: in ON rituality, preparing food is not implemented to reduce distress, as it occurs for OCD compulsions. Our results support the hypothesis according to which there is a relationship between worry’s contents, cognitive processes, and ON features. Unfortunately, the WDQ domains did not allow us to investigate worries related to health. Future studies should analyze health and healthy eating concerns, rather than general contents, differentiating them from typical pathological worry components to confirm this relation. Also, this study’s results do not entirely exclude the relationship with OC characteristics, which could be associated with or serve as a mediator of the orthorexic behavior. Future investigations could distinguish beliefs that underlie the compulsive behavior from those that underlie the orthorexic behavior.

In line with previous evidence [3, 36, 45], relationships between orthorexic characteristics and perfectionism traits are shown in the current study. Indeed, the MPS total score shows a positive correlation both with the EHQ total score and the EHQ “Knowledge”, “Problems”, and “Feelings” subscales. Above all, the non-adaptive perfectionism, characterized by high personal standards, concern over mistakes, and doubting of actions, continuously correlates with the ON features. Differences between High and Low EHQ are highlighted for the same subscales as well. If the obsessive symptomatology is controlled, these relationships remain substantially unchanged, and all perfectionism components come to light, except for the "Organization" MPS subscale. It is noteworthy that perfectionism is the only construct with a moderate relationship with the EHQ "Knowledge" subscale. Perfectionistic strivings could represent cognitive risk components for the subsequent development of orthorexic symptomatology as it happens in EDs [39].

Correlations with the BAI and the BDI-II are small and are all affected by the obsessive component, except for the EHQ "Feelings" subscale showing a significant correlation with the BAI that persists if the OCI-R effect is controlled. Between the two EHQ groups, covariation with the OCI-R highlights a significant difference as far as anxious and depressive components are concerned. The anxious/depressive component is more evident in the High EHQ group and becomes greater after controlling the OC symptoms. On the whole, it can be assumed that the obsessive component represents a mediating or collateral variable about the orthorexic symptomatology, and future studies will consider more accurate models of prediction of the orthorexic components to have an idea to better understand of the importance moderation role that each one has in the ON. Future studies should analyze health and healthy eating concerns, rather than general contents, differentiating them from typical pathological worry components (i.e., work, financial, and relationship issues) to confirm these relations. Besides, this study’s results do not entirely exclude the relationship with OC characteristics. Future investigations could distinguish beliefs that underlie the compulsive behavior from those that underlie the orthorexic behavior.

Future studies should investigate the relationship between ON and other mental disorders such as EDs, OCD, and GAD. Orthorexia Nervosa could be a prodromal syndrome, a paucisymptomatic form, or an outcome of a treatment. In the future, research on clinical populations of EDs in comparison with the general population may highlight the role of ON in EDs. Moreover, it would be important to assess beliefs or metacognitions related to OCD and GAD to understand their role in ON’s occurrence and maintenance.

## What is already known on this subject?

Scientific literature put in evidence a heterogeneous pattern of features implied in the ON conceptualization: some studies highlighted an overlap between EDs (especially, AN and BN), instead others have found a strong relation with OC and perfectionism features.

## What this study adds?

Based on our knowledge, this is the first study which investigated relations between ON and worry characteristics and its independence from OC symptomatology. Our study highlights that the obsessive component could represents a mediating or collateral variable in relation to the orthorexic symptomatology. In addition, a relationship between ON and Worry has been put in evidence.
